# Non-operative management of acute appendicitis in children

**DOI:** 10.1007/s00383-022-05284-y

**Published:** 2022-11-28

**Authors:** Soma Jumah, Tomas Wester

**Affiliations:** 1 Unit of Pediatric Surgery, Karolinska University Hospital, 17176 Stockholm, Sweden; 2 Department of Women’s and Children’s Health, Karolinska Institutet, Stockholm, Sweden

**Keywords:** Appendicitis, Children, Non-operative management

## Abstract

Appendicitis is one of the most common surgical emergencies in children and adults. Appendectomy as the standard care has been challenged in the recent years with growing evidence about non-operative treatment as a potential primary treatment in patients presenting with signs and symptoms suggestive of acute appendicitis. This review aims to establish where the recent research stands regarding conservative treatment of acute appendicitis, especially in children. There are several studies that report the potential safety and efficacy of treating acute appendicitis non-operatively. Several studies have challenged the concept of acute appendicitis being a progressive disease that always ends in perforation, rather than a disease that can present as different forms with only a defined number of cases progressing to perforation. The lack of randomized controlled studies is a limitation and well-designed randomized controlled trials are needed to determine the role of non-operative management of acute appendicitis in children.

## Introduction

Acute appendicitis and appendectomy as the standard care continues to be one of the most common surgical emergencies and procedures worldwide with peak incidence of acute appendicitis usually occurring in the second or third decade of life, [1] which makes it of significant relevance to children. It has been estimated that 1–8% of children presenting with acute abdominal pain at the emergency department get diagnosed with acute appendicitis [2].

The practice of treating acute appendicitis with surgery has recently been challenged due to controversy about it being a progressive disease and suggesting two disease entities of acute appendicitis; a mild inflammation that can settle spontaneously or with antibiotics and a more severe inflammation that can progress to gangrene and perforation, proposing the potential of developing different guidelines for the treatment of acute appendicitis depending on its form [3].

This review aims to touch down on the various aspects of the non-operative management of acute appendicitis in adults and more specifically in children based on previous randomized controlled trials (RCT) and review articles thus showing the possibilities of adopting new practices in treating acute appendicitis in children.

### Pathophysiology

The prevailing belief that appendicitis is caused by the bacterial infection and inflammation followed by luminal obstruction by an appendicolith has been challenged by the observation that an appendicolith is found only in a minority of acute appendicitis patients and that an appendicolith can be found in appendices without inflammation. However, luminal obstruction by an appendicolith, fibrous band, lymphoid hyperplasia or even a caecal carcinoma as a precursor to inflammation in acute appendicitis continues to be the proposed mechanism of pathogenesis, but there is growing interest in viral infections leading to secondary bacterial infection as a possible trigger.

A mix of aerobic and anaerobic bacteria, mostly *E. coli* and *Bacteroides spp.* have been isolated from removed inflamed appendices. Blunt abdominal trauma followed by vascular compromise has been also proposed. The difference of rates of acute appendicitis in different ethnicities, geographical regions and familial tendency can be a clue for a genetic predisposition, although no specific genes have been yet described. The environmental factors and diet and their interaction with genetic factors predisposing the individual to acute appendicitis remains unknown [1, 4].

The interaction between these different factors consequently leads to acute appendicitis that can be divided, based on macroscopic and microscopic appearances, into simple disease and complex disease. Simple appendicitis accounts for phlegmonous, non-perforated appendicitis. Complex appendicitis is usually a gangrenous or perforated appendix with or without abscess formation [1, 4].

### Spontaneous resolution of acute appendicitis

The theory that untreated appendicitis will eventually progress to perforation with an associated increase in mortality and morbidity has been accepted for years. Therefore, the standard practice for decades has been early exploration when suspicion of acute appendicitis exists. The justification for this has been the belief that perforation can be prevented and thus avoided if surgery is performed at the early stages of inflammation. However, this theory is being widely challenged. In a huge retrospective study that analysed data from over 56,000 patients found that the extent of appendectomy has an influence on the incidence rate of non-perforated appendicitis but not on the incidence rate of perforated appendicitis which suggests possible spontaneous recovery of acute appendicitis without increased risk for perforation [5]. Park et al. randomized 245 patients with CT-confirmed uncomplicated acute appendicitis to two groups, one group that received antibiotic treatment and one group that received supportive treatment without antibiotics and found no difference in treatment failure rates between the two groups [6]. Other studies have showed that a restrained attitude to exploration results in fewer patients with diagnosed appendicitis thus proposing spontaneous resolution as a possible destiny of the inflamed appendix [5].

During the COVID-19 pandemic, it was noted an overall reduction of appendicitis cases by 20.9% in adults and an increase of 13.4% in children. The rate of antibiotic treatment increased significantly, and higher rates of complicated appendicitis were observed in adults which can be explained by the overall decrease of the cases of appendicitis that was caused by lower numbers of patients with uncomplicated appendicitis seeking medical care, hence, spontaneously recovering, while the numbers of complicated appendicitis stayed the same [7].

### Operative management of acute appendicitis

The surgical removal of the inflamed appendix, appendectomy, has without doubt been the way of care and the gold standard in the treatment of acute appendicitis [8, 9]. Open surgery using McBurney’s incision remained the procedure of choice until the introduction of laparoscopy in 1983 [8]. Laparoscopy has many benefits over open surgery. The likelihood of post-surgical wound infections is reported to be reduced by at least half in laparoscopic appendectomy. Pain and the need for analgesia post-operatively was also reduced after laparoscopic surgery. Return of normal bowel function was faster after laparoscopic appendectomy. Lastly, laparoscopic appendectomy is also a diagnostic modality of great value especially in the cases of negative appendectomy [10].

### Non-operative management of uncomplicated acute appendicitis in adults

The possibility of finding an effective treatment modality with better complications profile, reduced cost, morbidity, and the promise of avoiding unnecessary surgery has led to expanding research that explores non-operative management of uncomplicated acute appendicitis [3].

In a study analysing mortality in patients who underwent appendectomy, it was surprisingly found that standardized mortality ratio increased ninefold after negative appendectomy with a discharge diagnosis of non-specific abdominal pain compared to 6.5-fold increased mortality after perforated appendicitis, hence pointing out that appendectomy is not a harmless operation [5].

Antibiotic therapy has been widely proposed as an effective and safe primary treatment for uncomplicated acute appendicitis [3, 11–13]. There is a growing number of systematic reviews and meta-analyses that compare antibiotics with surgery in acute uncomplicated appendicitis. Despite of the growing number of these articles, the results have to be considered with caution as the included RCTs have different inclusion criteria, exclusion criteria, and definition of outcomes, which can explain the different results achieved. Svensson et al. have done a meta-analysis based on per-protocol data; what the patient received as initial treatment rather than what they were randomised to receive, which showed no significant difference in treatment failure (defined as failure of non-operative treatment requiring appendectomy as well as incidence of negative appendectomies in those treated surgically) ratios between patients treated non-operatively versus those treated operatively. They also found that there were fewer complications in the group of patients treated conservatively. Interestingly, 73% of patients treated conservatively for suspected acute appendicitis did not require surgery during their initial admission or during 1-year follow-up [3].

At the 5-year-follow-up of the Appendicitis Acuta (APPAC) study, which was a multicentre, open label, noninferiority RCT, 61% of 256 patients presenting with uncomplicated acute appendicitis were successfully treated with antibiotics. Recurrence of acute appendicitis mostly occurred during the 1-year-follow-up and none of these patients suffered from complications related to delay of surgery [14, 15]. In a recent RCT done by Flum and colleagues, 1552 adults with acute appendicitis underwent randomization, 776 randomized to antibiotics and 776 to surgery. They, unlike previous studies, included patients with appendicolith, a condition usually associated with higher risk for perforation. Participants in the antibiotics group underwent surgery if they developed general peritonitis or septic shock or if their symptoms worsened after 48 h of the initiated therapy. They reported that antibiotics were non-inferior to appendectomy regarding 30-day health status assessed by the European Quality of Life Dimensions (EQ-5D). The incidence of appendectomy at 90 days in the antibiotics group was 29%. It is to be noted that 41% of the appendectomies were among those with an appendicolith and 25% among those without appendicolith. It was shown that complications were more common in the antibiotics group although this was mainly attributed to those having appendicoliths [12].

Andersson et al. divided patients presenting with abdominal pain suspected to be caused by acute appendicitis, clinically, based on appendicitis inflammatory response score (AIR score), into three groups. Low risk, intermediate risk, and high-risk groups. The results of this study demonstrated the safety, efficacy, and importance of risk stratification of patients presenting with symptoms suggestive of acute appendicitis and compared clinical assessment of those patients to the role of imaging. It also showed that imaging in patients with unclear diagnosis did not reduce hospital admissions or the number of negative appendectomies but was associated with an increase in the detection and treatment of potentially spontaneous resolving appendicitis. Above all, long-term follow-up did not report difference in appendectomy or appendicitis suggesting no increased risk of recurrence in patients with spontaneously resolving appendicitis [11].

All these findings draw the attention to the importance of the concept of detecting patients eligible for non-operative management including antibiotics treatment in any future research aiming to establish a conservative aspect as an alternative way in managing acute appendicitis [11].

### Non-operative management of acute uncomplicated appendicitis in children

In the recent years, several studies have been exploring the alternative of treating acute uncomplicated appendicitis in children conservatively. A feasibility trial done in the UK where they randomised children with suspected acute uncomplicated appendicitis based on clinical presentation while abstaining from doing any radiological assessment of the symptoms showed that treating acute uncomplicated appendicitis in children conservatively seem to be safe and feasible, which, in its turn, can pave the way for future RCTs. Despite the researchers’ effort to include only children with uncomplicated appendicitis, 30% of the children allocated to receive appendectomy had complicated appendicitis, which suggests that differentiating between uncomplicated and complicated appendicitis based only on clinical assessment is suboptimal.

Of the 11 children initially randomised to receive non-operative treatment, 41% had not undergone appendectomy by the end of the follow-up period. 24% of those randomised to non-operative treatment presented with recurrent appendicitis of which six children underwent appendectomy, four had histologically confirmed simple acute appendicitis while two had perforated appendicitis. One child presented with an appendix mass at the time of recurrence and was treated with antibiotics followed by interval appendectomy. They also showed that patients were able to be recruited outside of working hours keeping in mind that they held training sessions for medical personnel about the study and noticed that the recruitment rate increased after these sessions. This is of importance to any future RCTs in children because it can help increase awareness about the recent literature challenging the widespread dogma of surgery being the gold standard in the treatment of acute appendicitis and help lift sensitive questions regarding safety and child health [16]. This study goes in line with the findings of another pilot RCT, being the first of its kind, showing that treating acute uncomplicated appendicitis in children conservatively is attainable without concerns for safety as 92% of patients treated with antibiotics had initial resolution of symptoms. Recurrence of acute appendicitis during follow-up occurred in one patient, 5%. Overall, 62% of patients had not undergone an appendectomy during the initial 1-year follow-up period. The cohort was re-assessed in a 5-year follow-up study showing that at 5-year follow-up 46% of children treated for acute appendicitis had undergone an appendectomy although acute appendicitis was histologically confirmed in only 17%. Interestingly, none of the children previously treated conservatively re-presented with complicated appendicitis. It is worth mentioning that the authors used both clinical assessment and radiological imaging; ultrasound or computed tomography, in making the diagnosis of uncomplicated acute appendicitis to include patients [17, 18].

A recent meta-analysis analysed 21 studies with heterogenous methodology, including only 1 RCT, the pilot RCT by Svensson et al. It was reported that non-operative treatment of uncomplicated acute appendicitis in children is safe and efficient. 92% of the patients had resolution of symptoms during initial hospital stay. After discharge 16% of patients proceeded to appendectomy due to recurrent appendicitis or recurrent abdominal pain with normal appendix. It also showed that hospital stay and complications rate were similar in patients treated conservatively and those undergoing an appendectomy [19].

In another systematic review and meta-analysis done by Kessler et al., the results favoured appendectomy over conservative treatment regarding efficacy and showed reduced treatment efficacy and increased re-admission rate in patients treated conservatively. Outcomes, including less complications and lower re-admission rate as well as increased efficacy. Outcomes in patients who did not have an appendicolith were superior to those with an appendicolith suggesting that the presence of an appendicolith could compromise conservative treatment outcomes [20].

Georgiou et al. did a meta-analysis that analysed all articles reporting non-operative treatment for acute uncomplicated appendicitis in children and found initial treatment efficacy in 97% of children with acute uncomplicated appendicitis. The rate of recurrence was 14%. At the last follow-up, success rate evaluated by patients who did not need an appendectomy was 82%. Complications were similar in both groups, the conservatively treated and those who underwent appendectomy, but the length of hospital stay was shorter in children treated with appendectomy. Importantly they reported that major conclusions that can lead to change of practice cannot be withdrawn from this analysis because of the varying quality of the studies the analysis was based on. Many of the studies were of poor-quality with different inclusion criteria and different lengths of follow-up. However, the results can support the justification of future RCTs to further explore this way of treatment [2].

A meta-analysis done by Podda et al. that included studies comparing conservative treatment to surgical treatment of acute uncomplicated appendicitis in adults and children concluded that both surgery and antibiotic therapy can be regarded as primary management with the possibility of the patient being involved in the discussion and in deciding the treatment of choice. The study reported that conservative treatment had lower efficacy (8% treatment failure rate within 24–48, and an additional 20% might need a second hospitalization for recurrent appendicitis) than immediate surgery but that it was a safe option that did not increase the risk of perforation rate neither resulted in increased complications post-surgery [13].

### Non-operative management of complicated acute appendicitis in children

The management of complicated acute appendicitis in children usually consists of fluid resuscitation and treatment with broad spectrum antibiotics with controversy whether to proceed with immediate surgery or antibiotics as first-line treatment followed by interval appendectomy or no surgery at all. A recent meta-analysis by Vaos et al. included 15 studies of which two were RCTs and the other were non-randomised, retrospective or prospective studies. It concluded that complications rate and wound infection were significantly lower in patients treated conservatively compared to those treated with surgery, but the duration of hospital stay was shorter in the group of children treated surgically [21].

In a meta-analysis done by Duggan and colleagues two RCTs comparing early and interval appendectomy in children with perforated appendicitis were included and the authors divided patients with perforated appendicitis into two groups; one group presents patients with perforated appendicitis with abscess formation and another group that consists of patients with perforated appendicitis but without abscess formation. It was showed that outcomes of the different treatment modalities depended extensively on the subgroup of patients treated. Early appendectomy was reported to be favoured in children with perforated appendicitis with no abscess formation. However, in the subgroup of patients presenting with perforated appendicitis and intraabdominal abscess formation, the optimal treatment is still controversial. The study did not report any statistically significant differences between early and interval appendectomy in this subgroup of patients [22]. Fugazzola et al. showed in a recent meta-analysis that non-operative management in children with abscess or phlegmon was related to better results regarding complication and re-admission rate. However, children with free perforated appendicitis had lower complication profile and re-admission when treated operatively [23].

It has been debated if interval appendectomy is necessary. Children’s Interval Appendectomy (CHINA) study, was a multicentre, open label, RCT that addressed this research question. Children were randomised into two groups, one group that received antibiotics treatment and were followed up through outpatient clinics for 1 year, the other group received interval appendectomy done at a median of 66 days after treatment allocation. The recurrence rate of acute appendicitis was 12% in the active observation group. Interval appendectomy was associated with low complication rate (6%), but severe complications took place and one patient needed multiple surgeries. It also showed that conservative treatment was superior in this group of patients in means of length of hospital stay, the number of days needed to be back to normal activity, and it was also reported to be a more cost-effective approach. The authors also report results from a previous systematic review that estimated the risk of a carcinoid tumour in this population to be 1% which is within the range of overall incidence in the general population of developing a carcinoid tumour at any site [24].

A recent meta-analysis in adults presented that immediate surgical intervention may be particularly appropriate as a treatment option for complicated appendicitis in patients without a drainable collection. Despite the study’s conclusion that non-operative management of complicated acute appendicitis in adults had 25.7% failure rate and higher incidence for major bowel resection as well as higher morbidity compared to patients treated with acute appendectomy, it showed that all the 18 patients who underwent percutaneous drainage in the non-operative group were treated effectively non-operatively. Eight of these patients made it successfully to elective interval appendectomy and did not have symptoms at the time of the surgery and the remaining ten patients never underwent surgery [25]. This supports the idea of stratification of patients with complicated acute appendicitis into different disease categories and the possibility to optimize the treatment option based on that with complex disease presenting with an appendiceal mass (a phlegmon), an abscess or free perforation with peritonitis [23].

### Future studies

One aspect of the growing research about the non-operative treatment of acute appendicitis is its consequence on the length of hospital stay. Conflicting data have been reported in different studies. Many have concluded that hospital stay, both the initial hospitalization and the total length of stay, was longer in the initially conservatively treated group [14, 17, 21, 23]. While the CHINA trial showed shorter duration of hospital stay in patients treated with active observation compared to those who underwent interval appendectomy, [24] Simillis et al. reported no difference in the length of hospitalization between patients treated conservatively and those who underwent surgery, although heterogeneity between the studies analysed was reported [26]. Therefore, an interesting research question that has been contemplated upon in a recent study is the role of oral antibiotics vs. intravenous antibiotics in the management of acute appendicitis. Sippola et al. randomized patients with acute uncomplicated appendicitis confirmed by CT imaging into two groups. One group that received 7 days of oral moxifloxacin and the other group received 2 days intravenous ertapenem followed by 5 days of levofloxacin and metronidazole. They reported a 70.2% success rate, defined as discharge from hospital without need for surgery and absence of recurrent appendicitis within 1-year follow-up in the group of patients which received oral antibiotics alone versus 73.8% success rate in the group of patients who received intravenous antibiotics followed by oral antibiotics course. No statistically significant difference was reported between treatment groups regarding the length of hospital stay or sick leave. The study also delivered further evidence of the effectiveness and safety of antibiotics treatment in acute uncomplicated appendicitis [27].

Park et al. further challenged the discussion of treating acute uncomplicated appendicitis with randomising patients into two groups, one group that received antibiotics therapy for 4 days and another group that received only supportive therapy without antibiotics and found that treatment failure appeared to be the same in the two groups. The study was carried out at a single centre and the sample size was small. It was not sufficient for evidence to change common practice, but it showed that active observation is feasible and potentially effective compared to antibiotics therapy. More and larger RCTs that explore these dimensions of treatment are needed [6]. This approach is particularly interesting as the impact of treatment of acute appendicitis with antibiotics as well as appendectomy on the microbiome has been discussed. There is an ongoing randomised, double-blind, placebo-controlled, multicentre study to compare antibiotic therapy with placebo in the treatment of uncomplicated acute appendicitis (APPAC III) with a joint study that aims to collect rectal swabs as well as microbiological and histological samples from the removed appendix to explore the potential role of microbiological aetiology in the development of complicated appendicitis. Moreover, it aspires to evaluate immunological and microbiological factors involved in appendicitis recurrence after successful initial antibiotic therapy [9]. The common assumption that the appendix has no function but is rather a remanent of primordial oversized caecum for the digestion of cellulose has been widely disputed. Several studies have confirmed the role of the appendix as a commensal bacterial reservoir [28, 29]. The biofilm in the appendix is thought to protect its members from colonization with pathogens and can, therefore, help actively, knowing the appendix can function with anterograde peristalsis, with rehabilitating a healthy gut microflora following infection or antibiotic therapy [29]. The rate of appendectomies is higher in industrial countries which can interestingly be explained with the hygiene theory proposing a hyper reactivity of the immune system against commensal bacteria consequent to the absence of major infectious outbreaks in the gastrointestinal tract [28]. Interestingly, a study from China analysed gut microbiome using gene sequencing on faecal samples taken from healthy individual with prior appendectomy (HwA) and compared it to those taken from healthy individuals with no history of appendectomy (HwoA) and found that the gut bacterial composition of samples from HwA was less diverse than that of samples from HwoA and had a lower abundance of Roseburia, Barnesiella, Butyricicoccus, Odoribacter, and Butyricimonas species while HwA had higher gut fungi composition and diversity than HwoA [30].

## Conclusions

The previously established ground to the etiology, function, and management of acute appendicitis in adults and children have been widely challenged in recent literature with growing evidence supporting non-operative management of appendicitis as a potential treatment modality. More RCTs are needed to be define the role of non-operative management particularly in children. Research should also focus on developing strategies to differentiate the different forms of appendicitis in patients, which can allow patient stratification and furthermore tailoring of the recommended way of treatment for each patient. Future research should also focus on exploring the different mechanisms of inflammation and infection behind the different forms of acute appendicitis, which can allow better understanding of the disease pathophysiology and course.

## Data Availability

Original data are available on request.
